# The efficacy of sumac (*Rhus coriaria *L.) powder supplementation in biochemical and anthropometric measurements in overweight or obese patients with non-alcoholic fatty liver disease: A double-blind randomized controlled trial

**DOI:** 10.22038/AJP.2024.23927

**Published:** 2024

**Authors:** Mohsen Mohit, Mohammadali Nejati, Najmeh Hejazi, Shayan Modaresi

**Affiliations:** 1 *Student Research Committee, Department of Clinical Nutrition, School of Nutrition and Food Sciences, Shiraz University of Medical Sciences, Shiraz, Iran*; 2 *Department of Internal Medicine, Gastroenterohepatology Research Center, School of Medicine, Shiraz University of Medical Sciences, Shiraz, Iran*; 3 *Department of Clinical Nutrition, School of Nutrition and Food Sciences, Shiraz University of Medical Sciences, Shiraz, Iran*

**Keywords:** Non-alcoholic fatty liver, Rhus coriaria, Sumac

## Abstract

**Objective:**

This study aimed to assess the effect of sumac supplement in biochemical and anthropometric measurements in overweight or obese patients with non-alcoholic fatty liver disease.

**Materials and Methods:**

In this double-blind randomized controlled trial, 45 NAFLD patients were randomly divided into two groups. The intervention group received sumac capsules (3 g/day) with a balanced diet for 8 weeks, while the placebo group received placebo with a balanced diet. Anthropometric indices, lipid profile, fasting blood glucose, insulin, Homeostatic Model Assessment for Insulin Resistance, aspartate transaminase, alanine aminotransferase, high sensitivity C-reactive protein and malondialdehyde were measured at baseline and at the end of the study.

**Results:**

The results revealed a significant decrease in anthropometric indices (weight (p=0.001), body mass index (p=0.001), waist circumference (p=0.001), body fat mass (p=0.001), body fat percentage (p=0.001), visceral fat score (p=0.001), biochemical levels of total cholesterol (p=0.007), fasting blood sugar (p=0.006), insulin (p=0.004) and HOMA-IR (p=0.002)) after the intervention compared to the baseline. However, no significant difference was observed between the two groups concerning anthropometric and biochemical indices.

**Conclusion:**

In this study, no significant differences were observed between the two groups regarding anthropometric and biochemical indices. Thus, further studies with larger sample sizes are recommended to be conducted on the issue.

## Introduction

Non-Alcoholic Fatty Liver Disease (NAFLD) is the most common liver metabolic disorder whose incidence is directly linked to cardiovascular disease and diabetes (Musso et al., 2011; Blachier et al., 2013). It can progress from a simple to the most complex liver disease. According to the estimates in Western countries, NAFLD will be the main cause of liver transplantation by 2030. Patients with NAFLD often do not have any specific symptoms. Some of the symptoms include weakness, fatigue, and vague pain, making it difficult to diagnose NAFLD in the early stages. In other words, this disease progresses without alarming the patients (Younossi et al., 2016).

Although there is no conclusive consensus on the pathogenesis of NAFLD, the "two hit" hypothesis has attracted great attention. The first hit is impaired lipid homeostasis and accumulation of triglycerides and free fatty acids in liver cells (steatosis). Lipogenesis can also occur due to increased insulin resistance in these patients. In the second hit, the inflammatory condition prevails and it is identified by the acceleration of oxidative stress, pro-inflammatory cytokine production, and mitochondrial defect, leading to progression to more complex stages of the disease (Day et al., 1998). 

The high prevalence of NAFLD, insufficient attention to its treatment, and lack of approved medications have increased the importance of this disease (Younossi et al., 2016). At present, lifestyle modification including weight loss and physical activity is the main strategy for the treatment of patients with NAFLD (Thoma et al. 2012; Rinella et al., 2015). 

The usage of medicinal plants for the treatment of diseases has been of interest since old times. Extensive usage of sumac as a tasty condiment in Iran, Turkey, and other countries has attracted researchers’ attention (Rayne et al., 2007; Chakraborty et al., 2009). Sumac is classified in the *Rhus* genus and flowering plant species from the Anacardiaceae family (Rayne et al., 2007). It is one of the native plants of Iran, Turkey, and Mediterranean countries. The flowers of this bush have clusters that change into small red spherical fruits (Chakraborty et al., 2009). Sumac contains tannins, phenolic acid, gallic acid, and quercetin. Some studies have reported the antimicrobial, antioxidant, anti-inflammatory, anti-diabetic, anti-atherosclerotic*,* and hypolipidemic effects of sumac powder (Shabana et al., 2011; Capcarova et al., 2012).

Obesity plays an important role in exacerbating inflammation and oxidative stress in hepatocytes. Previous studies indicated the lipase inhibitory (Jaradat et al., 2017) and anti-obesity effects (Heydari et al., 2019; Hariri et al., 2020) of sumac powder. *In-vitro* studies also showed that sumac powder could protect hepatocytes against oxidative stress by reducing Reactive Oxygen Species (ROS) production and lipid peroxidation, and regulation of glutathione in the mitochondrial membrane (Pourahmad et al., 2010). This may illustrate sumac powder as a novel alternative herbal medicine in the treatment of patients with NAFLD. 

To the best of our knowledge, no clinical trial has evaluated the effect of sumac powder among patients with NAFLD. Therefore, the present trial aimed at assessing the effect of sumac powder on biochemical and anthropometric parameters in overweight/obese patients with NAFLD.

## Materials and Methods

**Ethics**

This double-blind, randomized, placebo-controlled clinical trial was done in concordance with the Declaration of Helsinki and good clinical practice guidelines. Additionally, the study protocol was checked and approved by the local Ethics Committee of Shiraz University of Medical Sciences (IR.SUMS.REC.1398.914). It was also registered in the Iranian Registry of Clinical Trials (IRCT20191009045043N1).

**Participants**

To identify the eligible participants, patients with NAFLD who referred to the Liver and Gastroenterology Clinic of Shahid Motahari Polyclinic affiliated to Shiraz University of Medical Sciences, Shiraz, Iran, were screened from January to April 2020. The inclusion criteria of the study were aging 20-60 years, diagnosis of NAFLD via ultrasound imaging approved by a physician, Body Mass Index (BMI) = 25-35 kg/m^2^, and being able to manage life without help. The patients with a history of alcohol consumption, liver disorders (cancer, hepatitis, or hereditary), or kidney, cardiovascular, lung, or thyroid diseases were excluded from the study. Pregnant or lactating women, patients undergoing obesity surgeries or weight loss diets, or those taking any herbal/biochemical medicines affecting liver function (ursodeoxycholic acid, phenytoin, amoxicillin, or lithium) or any medical treatment for NAFLD and any dietary supplements (fiber, omega-3, and antioxidants) in the past three months were also excluded from the study.

**Sample size**

Considering the decrease in the level of Low Density Lipoprotein-Cholesterol (LDL-Chol) in Sabzghabaei et al. study (Sabzghabaee et al., 2014) and based on the mean difference of 131, Standard Deviation (SD) of 31.9, power of 80%, and α=0.05, the final sample size By considering 15% dropouts was calculated 23 patients per group*. *

**Study design**

**Figure 1:**
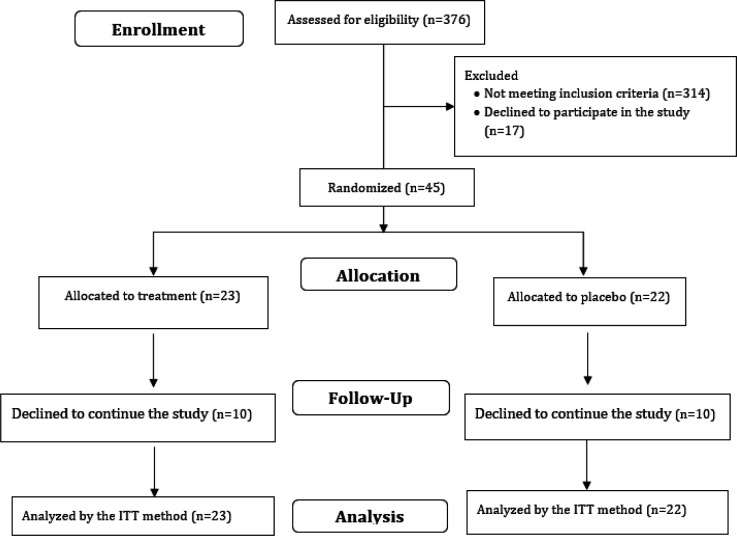
Consolidated standards of reporting trials (CONSORT) flow diagram

After screening 376 patients, 45 eligible ones were selected to participate in the study (Consort diagram, Figure 1).

The study participants were informed about the study protocol and their consent forms were obtained. Then, the random allocation software was used to randomize the participants into two equal groups (treatment and control) by block randomization with fixed block size of two. The participants in the treatment group received 3 g/day sumac powder (six 500-mg capsules, two capsules after each meal), while the control group patients received 3 g/day rice flour as placebo (six 500-mg capsules, two capsules after each meal) for eight weeks. In this study, in order to prepare sumac powder, fresh sumac in the clusters form was collected from Qalat destrict (Zagros forest), Fars, Iran. Sumac clusters were dried in the shade and then the fruit were separated from the clusters. The sumac fruits were separated with a sieve and ground. Sumac powder and placebo encapsulation was done in the school of pharmacy, shiraz University of Medical Sciences. Also, a sample of the sumac clusters (which was used in this study) was identified by Mrs Sadigheh Khademian (Department of Traditional Pharmacy, Shiraz University of Medical Sciences, Shiraz, Iran) and registered in the herbarium of School of pharmacy, Shiraz University of Medical Sciences. (number: PM1321*-Rhus coriaria *L.). All the patients in sumac and placebo groups also received an individualized diet. The estimated energy intake of diets was determined by ideal body weight (BMI=21.5-23 kg/m^2^) with a 500 kcal/day calorie deficit. The calories distribution was as follows: 55% carbohydrate, 17% protein, and 28% fat.

**Measurements**

At the beginning of the study, a demographic questionnaire was completed by the participants. The International Physical Activity Questionnaire (IPAQ) and a 24-hr dietary recall for three days were also recorded for each participant at the beginning and at the end of the study. The patients’ weight (kg) and height (cm) were measured by a Seca scale (to the nearest 0.1 kg and 0.1 cm, respectively) at baseline and at the end of the study. Then, BMI was calculated by dividing weight (kg) by height (m^2^). In addition, body composition parameters including fat mass, fat-free mass, and visceral fat score were evaluated in standing position by bioimpedance analysis (BIA, Tanita) at the beginning and end of the study. Other anthropometric measures including waist circumference (cm), hip circumference (cm), and waist-to-hip ratio were also recorded at the beginning of the study and eight weeks after the intervention. Moreover, 5 ml venous blood was collected from each participant after a 10-12-hr fasting before and after the intervention. The blood samples were centrifuged at 200 rpm/min for 10 min and the sera were frozen at -70˚C until analysis. Lipid profile (triglycerides, total cholesterol, LDL-Chol, and High-Density Lipoprotein-Cholesterol (HDL-Chol)), fasting blood sugar, aspartate transaminase (AST), and alanine aminotransferase (ALT) were measured by an autoanalyzer (BT-1500 autoanalyzer using Pars Azmoun kits, Iran). Serum levels of insulin and high sensitivity C-reactive protein (hs-CRP) were also determined by ELISA kits (LDN, Nordhorn, Germany). Besides, the serum level of malondialdehyde (MDA) was assessed by measuring Thiobarbituric Acid Reactive Substances (TBARS) using a spectrophotometric assay. Finally, Homeostatic Model Assessment for Insulin Resistance (HOMA-IR) was determined using the following formula: [fasting glucose(mg/dl)]$\times [\mathrm{fasting insulin}(\frac{\mu U}{\mathrm{ml}})]/405$ (Salgado et al., 2010).

**Statistical analysis**

Data analysis was done via the intention-to-treat method using the SPSS 19 software. Shapiro-Wilk test was used to determine the normal distribution of the data. Quantitative data is presented as median (interquartile range, and 25^th^ and 75^th^ quartiles), while quantitative data is presented as number (percentage). Mann-Whitney U-test and Wilcoxon signed ranks were used for between-group and within-group comparisons, respectively. A p<0.05 was considered statistically significant. 

## Results

This study was conducted on 45 eligible patients with NAFLD. However, ten patients in the sumac group and ten patients in the placebo group did not complete the study. The co-occurrence of the COVID-19 pandemic and the second phase of data collection at the end of the study was the main reason for the participants’ reluctance to complete the study (Figure 1).

The baseline characteristics of the participants in both study groups are presented in Table1. The results revealed no significant difference between the two groups in terms of age, gender, weight, BMI, or physical activity level at the baseline. 

**Table 1:** Baseline characteristics in the sumac and placebo groups

p-value ^a^	Placebo group (n=22)	Sumac group (n=23)	Variable
0.570	46.00 (34.00 – 54.25)	45.00 (40.00 – 56.00)	Age (years)
0.884 ^b^	11 (50%) 11 (50%)	12 (52.2%) 11(47.8%)	Sex; frequency (%) Male Female
0.433	30.84 (75.20 – 89.25)	78.80 (73.20 – 87.40)	Weight (kg)
0.709	3.14 ± 29.17	3.00 ± 29.51	Body mass index (kg/m^2^)
0.334	274.82 (160.71 – 362.67)	318.21 (197.67 – 569.28)	Physical activity (MET- min/week)
0.722	109.13 ± 17.64	107.17 ± 19.01	FBS (mg/dl(
0.770	15.55 ± 13.27	15.54 ± 9.55	Fasting insulin (μIU/ml)
0.619	4.49 ± 4.53	3.93 ± 3.03	HOMA-IR
0.865	20.00 (14.75 – 25.00)	19.00 (16.00 – 23.00)	AST (IU/L)
0.142	31.18 ± 19.10	24.20 ± 11.35	ALT (IU/L)
0.558	103.00 ± 24.61	107.21 ± 23.27	LDL-C (mg/dl)
0.436	39.86 ± 7.39	41.69 ± 8.17	HDL-C (mg/dl)
0.847	179.50 (142.00 – 222.50)	173.00 (119.00 – 241.00)	Triglyceride (mg/dl)
0.445	186.00 (158.00 – 221.50)	195.00 (175.00 – 221.00)	Cholesterol (mg/dl)
0.725	3.56 (2.16 – 5.22)	2.85 (2.22 – 5.90)	MDA (μm)
0.153	1761.74 (1429.59 – 2791.56)	2302.82 (1499.34 – 3226.54)	Hs-CRP (pg/ml)

p<0.05 was considered statistically significant. Abbreviations: LDL-C, low-density lipoprotein cholesterol; HDL-C, high-density lipoprotein cholesterol; AST, aspartate aminotransferase; ALT, alanine transaminase; FBS, fasting blood sugar; HOMA-IR, homeostatic model assessment for insulin resistance; MDA, malondialdehyde; Hs-CRP, high-sensitivity C-reactive protein. The data is expressed as median [IQR] and mean±SD for nonparametric and parametric data, respectively. ^a^ Comparison of the two groups by independent samples t*-*test (for parametric data) and Mann–Whitney U test (for nonparametric data).^ b ^Obtained from chi-square test.

The results revealed no significant difference between the two groups regarding the changes in the dietary intake (energy, macronutrients, and some micronutrients) during the study. However, significant changes were observed within the study groups after the intervention compared to the baseline (p<0.05)(Table 2).

The anthropometric parameters before and after the intervention are listed in Table 3. Accordingly, a significant decrease was observed in weight, BMI, waist circumference, body fat mass, body fat percentage, and visceral fat in both groups during the study. However, the differences between the study groups regarding those measurements were not significant after the intervention. Based on the results presented in Table 4, the serum levels of cholesterol and fasting insulin decreased significantly in the sumac group at the end of the study. In addition, glycemic indices including fasting blood sugar and HOMA-IR reduced significantly within both groups eight weeks after the intervention. However, no significant difference was observed between the two groups with respect to the biochemical parameters during the study.

**Table 2:** Dietary intake and physical activity in the sumac and placebo groups

Variables		Sumac group (n=23)			Placebo group (n=22)			p-value^b^
		Before	After	p-value^a^	Before	After	p-value^a^	
Energy (Kcal/day)	1897.70 (1340.63–2325.00)		1468.00 (1184.31–2298.95)	0.003	1833.99 (1320.07–2610.50)	1747.29 (1306.76–2548.25)	0.002	0.900
Carbohydrate (g/day)	271.51 (210.39–348.20)		253.80 (193.93–324.90)	0.013	294.9 (239.48–393.25)	260.71 (225.37–352.35)	0.004	0.802
Protein (g/day)	71.07 (48.77–100.20)		52.21 (45.60–94.09)	0.003	82.2 (53.52–110.57)	71.12 (53.52-100.29)	0.015	0.257
Fat (g/day)	45.83 (22.45–69.81)		45.25 (22.11–69.80)	<0.001	47.58 (27.59–86.74)	41.56 (25.92-77.40)	0.006	0.243
Dietary fiber (g/day)	26.09 (19.43–32.74)		26.09 (18.72–30.03)	0.008	29.82 (24.58–33.68)	29.73 (19.58–35.04)	0.463	0.293
Vitamin E (mg/day)	3.79 (1.91-4.97)		2.47 (1.92–3.90)	0.016	29.82 (24.58–33.68)	29.73 (19.58–35.04)	0.463	0.658
Physical activity (MET-min/ week)	318.21 (197.67–569.28)		318.21 (234.64-548.57)	0.595	274.82 (160.71–362.67)	207.10 (185.65–452.33)	0.171	0.382

The data is presented as median [IQR]. p<0.05 was considered statistically significant. ^a ^Obtained from Wilcoxon test. ^b ^Obtained from Mann–Whitney test.

**Table 3:** Anthropometric parameters in the sumac and placebo groups

Variables	Sumac group (n=23)			Placebo group (n=22)			p-value^b^
	Before	After	p-value^a^	Before	After	p-value^a^	
Weight (Kg (	78.80 (73.20 – 87.40)	77.60 (72.60 – 83.50)	0.001	84.10 (75.20 – 89.25)	84.30 (72.17 – 87.75)	0.023	0.432
Body mass index (Kg/m^2^)	29.40 (27.69 – 31.10)	28.70 (26.77 - 31.10)	0.001	29.00 (27.17 – 30.47)	28.57 (27.07 – 29.65)	0.002	0.626
Waist circumference (Cm)	102.00 (96.00–108.00)	0.98 (93.00–108.00)	0.001	101.00 (96.75 – 105.75)	99.50 (96.75 – 105.75)	0.002	0.401
Waist to hip ratio	0.97 (0.95 -1.01)	0.98 (0.95 – 1.00)	0.254	0.95 (0.91 – 0.98)	0.96 (0.92 – 0.99)	0.171	0.981
Body fat (Kg)	26.30 (22.90 – 30.30)	25.80 (21.30 – 27.60)	0.001	26.15 (19.97 – 31.15)	24.58 (19.97 – 31.02)	0.002	0.357
Body fat percentage (%)	32.80 (28.10 – 30.38)	32.40 (28.10 – 36.10)	0.001	31.65 (24.57 – 36.50)	30.05 (24.12 – 35.60)	0.008	0246
Free fat mass (kg (	52.20 (45.90 – 64.10)	52.30 (46.20 – 60.80)	0.196	53.25 (46.37 – 64.12)	54.75 (44.12 – 64.11)	0.483	0.083
Visceral fat score	9.00 (8.00 – 12.00)	8.00 (8.00 – 12.00)	0.002	8.00 (7.00 – 10.50)	7.00 (6.75 – 10.25)	0.001	0.887

The data is presented as median [IQR]. p<0.05 was considered statistically significant.^a ^Obtained from Wilcoxon test.^b ^Obtained from Mann–Whitney test.

**Table 4:** Biochemical markers of the patients in the sumac and placebo groups

Variables	Sumac group (n=23)			Placebo group (n=22)			
	Before	After	p-value^a^	Before	After	p-value^a^	p-value^b^
Triglyceride (mg/dl)	150.00 (120.00 – 230.00)	173.00 (119.00– 241.00)	0.084	179.50 (142.00 – 222.50)	151.00 (119.75-189.25)	0.263	0.821
Cholesterol )mg/dl(	195.00 (175.00 – 221.00)	189.00 (159.00– 214.00)	0.007	186.00 (158.00- 221.50)	190.00 (167.50-207.50)	0.556	0.103
LDL-C )mg/dl(	107.00 (93.00 – 123.00)	107.00 (82.00 – 112.00)	0.169	99.50 (81.50 – 118.75)	107.00 (90.50 – 119.75)	0.754	0.276
HDL-C (mg/dl)	40.00 (35.00 – 45.00)	40.00 (34.00 – 46.00)	0.38	38.50 (35.50 – 43.25)	38.50 (36.00 – 46.00)	0.182	0.125
AST (IU/L)	19.00 (16.00 – 23.00)	18.00 (15.00 – 22.00)	0.138	20.00 (14.75 – 25.00)	19.50 (12.75 – 26.00)	0.964	0.208
ALT (IU/L)	23.00 (16.00 – 30.00)	23.00 (16.00- 29.00)	0.875	31.50 (13.25 – 42.55)	23.50 (13.25 – 49.00)	0.844	0.728
FBS (mg/dl)	104.00 (95.00 – 120.00)	101.00 (87.00 – 113.00)	0.006	107.00 (95.50 – 126.50)	103.00 (94.00 – 110.50)	0.018	0.971
Fasting insulin IU/ml(μ)	12.20 (6.20 – 20.60)	11.40 (5.30 – 19.10)	0.004	11.25 (7.05 – 20.77)	11.90 (5.07 – 17.40)	0.182	0.264
HOMA-IR	3.03 (1.31 – 5.91)	2.85 (2.22 – 5.90)	0.002	2.82 (2.03 – 6.21)	2.82 (1.21 – 4.73)	0.019	0.422
MDA (MOLμ)	2.85 (2.22 – 5.90)	3.18 (1.92 – 4.24)	0.281	3.56 (2.16 – 5.22)	2.95 (2.03 – 5.00)	0.177	0.980
Hs-CRP (pg/ml)	2302.82 (1499.34– 3226.54)	1999.23 (14.10– 2720.34)	0.311	1761.74 (1429.59–2791.56)	1819.54 (1429.59-2991.5)	0.084	0.101

The data is presented as median [IQR]. Abbreviations: LDL-C, low-density lipoprotein cholesterol; HDL-C, high-density lipoprotein cholesterol; AST, aspartate aminotransferase; ALT, alanine transaminase; FBS, fasting blood sugar; HOMA-IR, homeostatic model assessment for insulin resistance; MDA, malondialdehyde; Hs-CRP, high-sensitivity C-reactive protein. p<0.05 was considered statistically significant. a Obtained from Wilcoxon test. b Obtained from Mann–Whitney test

## Discussion

The present study was a randomized, double-blind, placebo-controlled clinical trial. To the best of our knowledge, this was the first study to evaluate the effect of sumac powder amongst overweight/obese patients with NAFLD. The results revealed a significant decrease in weight, BMI, waist circumference, body fat mass, body fat percentage, visceral fat score, biochemical levels of total cholesterol, fasting blood sugar, and fasting insulin, and HOMA-IR in the group that received 3 g/day of sumac powder along with a Reduced Calories Diet (RCD). However, there was no statistically significant difference between the two groups regarding these parameters.

In this study, a diet with a similar calorie deficit (i.e. 500 kcal) was used in both groups. The results indicated a significant decrease in energy and macronutrient intake in both groups at the end of the intervention, which led to a significant reduction in anthropometric parameters including weight, BMI, waist circumference, body fat percentage, and visceral fat score in both groups. This showed the participants' adherence to the study protocol as well as the effectiveness of the prescribed diet in both groups. Nevertheless, the differences between the two groups were not statistically significant. These results were in agreement with those of the studies carried out by Ardakani et al. on patients with type II diabetes and Ardalani et al on hypertensive patients (Ardalani et al., 2016; Fatahi et al., 2016), but were in contrast with those of the studies performed on hyperlipidemic and obese groups (Asgary et al., 2018; Hariri et al., 2020). The discrepancy between the findings might be attributed to the type of sumac, sample size, duration of the intervention, the dosage used, method of sumac administration, and participants’ health status.

Although liver enzymes alone are insufficient to assess the severity of liver damage and are needed to be combined with other diagnostic methods such as ultrasonography, elevated levels of these enzymes in patients with NAFLD originate from inflammation and a defective cycle which can result in a rising trend in inflammation and oxidative stress. The present study findings indicated a decreasing trend in AST and ALT levels in both groups at the end of the study. *In-vitro* studies have referred to many hepatoprotective effects for sumac powder (Pourahmad et al., 2010; Anwer et al., 2013; Salimi et al., 2015). Nonetheless, human evidence is limited and conflicting in this context. The type of sumac used in various studies and how it is processed lead to a significant difference in the antioxidant composition of sumac, which can be the main reason for the difference in the results related to the antioxidant properties of sumac. In line with the current investigation, HajMohammadi et al. revealed no significant difference in AST and ALT levels after six weeks of supplementation with 1000 mg/day sumac powder (Hajmohammadi et al., 2018). On the contrary, Kazemi et al. reported a significant decrease in AST and ALT levels after 12 weeks of supplementation with 2000 mg/day sumac powder (Kazemi et al., 2020).

The current study findings indicated a significant decrease in fasting blood sugar, insulin, and HOMA-IR in the intervention group and a significant decline in fasting blood sugar and HOMA-IR in the placebo group. However, there were no significant differences between the two groups concerning the mentioned indicators. A recent meta-analysis also demonstrated that the restricted calorie diet could decrease fasting blood sugar, insulin, and HOMA-IR. *In-vitro* studies have attributed the inhibitory effects of pancreatic alpha-amylase and alpha-glucosidase, inhibition of SREP1 gene expression, and increased expression of GLUT4 and PPAR-Y genes to sumac (Giancarlo et al., 2006; Mohammadi et al., 2010; Chen et al., 2016; Belwal et al., 2017). However, contradictory results were obtained in human trials. Two randomized controlled trials reported no significant differences in glycemic indices (Salimi et al., 2011; Shidfar et al., 2014), while three trials indicated significant differences in glycemic indices after the intervention using sumac (Anwer et al., 2013; Fatahi et al., 2016; Asgary et al., 2018).

Dyslipidemia is one of the most common disorders in patients with NAFLD, which results from impaired lipid homeostasis by the liver. Cohort studies have shown that a significant percentage of people with cardiovascular diseases have fatty liver (Ekstedt et al., 2006). The present study results revealed a significant decrease in the serum level of cholesterol in the sumac group. However, no significant difference was found between the two groups regarding the lipid profile. In this context, contradictory results have been obtained in different trials, which could be associated with the differences in the participants’ initial blood lipid levels as well as their health status (Sabzghabaee et al., 2014; Asgary et al., 2018; Hajmohammadi et al., 2018).

The development of inflammatory and oxidative stress conditions theoretically dominate at the second stage of the "two hit" hypothesis. These conditions damage hepatocytes, which leads to the disease progression to cirrhosis and fibrosis of the liver tissue. Many studies have emphasized that patients with NAFLD are at a higher risk of cardiovascular death. The present study results indicated no statistically significant difference between the two groups regarding hs-CRP or MDA levels. Moreover, the results revealed a decrease in vitamin E intake along with reduced food intake, especially in the sumac group. Vitamin E is a powerful antioxidant that has been reported to have beneficial anti-inflammatory and anti-oxidative stress effects on the treatment of NAFLD. A meta-analysis demonstrated that vitamin E supplementation improved biochemical, inflammatory, and hepatic fibrosis parameters (Sato et al., 2015). The lack of significant changes in inflammatory factors and oxidative stress in the present study could be attributed to the reduced dietary intake of vitamin E.

The strengths of this study were investigating multiple biochemical factors and considering a double-blind design. However, one of its major limitations was the drop in sample size due to the co-occurrence of the post-intervention phase with the onset of the COVID-19 pandemic. Further studies are recommended to assess the effect of geographical differences on the properties of sumac and to determine the impact of reducing the intake of effective factors alongside a restricted calorie diet on the treatment of NAFLD.

In this study, consumption of 3 g/day sumac powder by obese/overweight patients with NAFLD for eight weeks did not significantly affect the biochemical and anthropometric parameters compared to the placebo group. Thus, future studies with larger sample sizes and longer intervention durations are recommended to be conducted on the issue.
